# Left ventricular global longitudinal strain in bicupsid aortic valve patients: head-to-head comparison between computed tomography, 4D flow cardiovascular magnetic resonance and speckle-tracking echocardiography

**DOI:** 10.1007/s10554-020-01883-9

**Published:** 2020-05-25

**Authors:** Allard T. van den Hoven, Sultan Yilmazer, Raluca G. Chelu, Roderick W. J. van Grootel, Savine C. S. Minderhoud, Lidia R. Bons, An M. van Berendoncks, Anthonie L. Duijnhouwer, Hans-Marc J. Siebelink, Annemien E. van den Bosch, Ricardo P. J. Budde, Jolien W. Roos-Hesselink, Alexander Hirsch

**Affiliations:** 1 Department of Cardiology, Erasmus MC, University Medical Center Rotterdam, Room Rg-419, P.O. Box 2040, 3000 CA Rotterdam, The Netherlands; 2 Department of Radiology and Nuclear Medicine, Erasmus MC, University Medical Center Rotterdam, Rotterdam, The Netherlands; 3 Department of Cardiology, Radboud University Medical Center Rotterdam, Rotterdam, The Netherlands; 4 Department of Cardiology, Leiden University Medical Center Rotterdam, Rotterdam, The Netherlands

**Keywords:** Echocardiography, Computed tomography, Cardiac magnetic resonance imaging, 4D flow, Global longitudinal strain, Bicuspid aortic valve

## Abstract

Left ventricular global longitudinal strain (LVGLS) analysis is a sensitive measurement of myocardial deformation most often done using speckle-tracking transthoracic echocardiography (TTE). We propose a novel approach to measure LVGLS using feature-tracking software on the magnitude dataset of 4D flow cardiovascular magnetic resonance (CMR) and compare it to dynamic computed tomography (CT) and speckle tracking TTE derived measurements. In this prospective cohort study 59 consecutive adult patients with a bicuspid aortic valve (BAV) were included. The study protocol consisted of TTE, CT, and CMR on the same day. Image analysis was done using dedicated feature-tracking (4D flow CMR and CT) and speckle-tracking (TTE) software, on apical 2-, 3-, and 4-chamber long-axis multiplanar reconstructions (4D flow CMR and CT) or standard apical 2-, 3-, and 4-chamber acquisitions (TTE). CMR and CT GLS analysis was feasible in all patients. Good correlations were observed for GLS measured by CMR (− 21 ± 3%) and CT (− 20 ± 3%) versus TTE (− 20 ± 3%, Pearson’s r: 0.67 and 0.65, p < 0.001). CMR also correlated well with CT (Pearson’s r 0.62, p < 0.001). The inter-observer analysis showed moderate to good reproducibility of GLS measurement by CMR, CT and TTE (Pearsons’s r: 0.51, 0.77, 0.70 respectively; p < 0.05). Additionally, ejection fraction (EF), end-diastolic and end-systolic volume measurements (EDV and ESV) correlated well between all modalities (Pearson’s r > 0.61, p < 0.001). Feature-tracking GLS analysis is feasible using the magnitude images acquired with 4D flow CMR. GLS measurement by CMR correlates well with CT and speckle-tracking 2D TTE. GLS analysis on 4D flow CMR allows for an integrative approach, integrating flow and functional data in a single sequence. Not applicable, observational study.

**Electronic supplementary material:**

The online version of this article (10.1007/s10554-020-01883-9) contains supplementary material, which is available to authorized users.

## Introduction

For decades left ventricular (LV) ejection fraction (EF) has been the gold standard for quantification of systolic LV function [1]. It has been a key metric in therapy and prognostication, in particular in patients with valvular heart disease. However, more sensitive methods have since been in development; [2] of which LV global longitudinal strain (GLS) is currently accepted as a more sensitive measurement, that may already be reduced before a decrease in LV EF can be observed. Moreover LV GLS allows for quantitative assessment of global and segmental ventricular function by measuring myocardial deformation, largely independent of angle and ventricular geometry [3–5]. GLS is defined as the percentage of shortening between the end-diastolic and end-systolic length of the myocardium. This technique of deformation measurement has been validated in different populations using speckle-tracking echocardiography [5–15]. More recently it was shown that GLS can also be derived from multiphase Computed Tomography (CT) datasets and conventional Cardiovascular Magnetic Resonance (CMR) steady state free-precession (SSFP) cine imaging using feature-tracking algorithms [16, 17]. However, these techniques, especially GLS measurement using CT are still new and not yet very well validated. In this study we propose a novel method that uses this feature-tracking algorithm on magnitude images acquired during 4D flow CMR to quantify LV volumes and GLS. 4D flow CMR allows for comprehensive post-hoc evaluation of blood flow patterns by 3D blood flow visualization and quantification of flow parameters [18]. Previous studies have shown that quantification of ventricular volume and function can be accomplished with 4D flow MRI with precision and inter-observer agreement comparable to that of SSFP cine imaging [19, 20]. Strain analysis would be a valuable additional feature of 4D flow CMR, as this would allow for integrative analysis of flow and function in one sequence.

The aim of this study was to assess the feasibility of left ventricular global longitudinal strain (LVGLS) measurement using magnitude 4D flow Cardiovascular Magnetic Resonance (CMR) and dynamic computed tomography (CT) datasets, and to provide data on the correlation between these novel approaches and the ‘gold standard’ of speckle-tracking derived GLS values using two-dimensional echocardiography (TTE).

## Methods

In this prospective cohort study, adult patients with a bicuspid aortic valve (BAV) were included [21–23] The study protocol consisted of TTE, CT and CMR on the same day. The inclusion criteria were age ≥ 18 year and one of the following: [1] aortic stenosis (gradient > 2.5 m/s), [2] aortic regurgitation (at least moderate) or [3] ascending aortic dilation ≥ 40 mm and/or aortic size index > 2.1 cm/m^2^. Patients with contra-indications to CT, CMR or contrast agents were excluded. For the current study we only included patients who underwent at least two of the three imaging modalities. The study complied with the Declaration of Helsinki and was approved by the medical ethical committee of the Erasmus Medical Center (MEC14-225). Written informed consent was provided by all patients.

### Echocardiography

One of two experienced sonographers performed a standard two-dimensional transthoracic echocardiogram. All studies were acquired using harmonic imaging on an EPIQ7 ultrasound system (Philips Medical Systems, Best, the Netherlands) equipped with an × 5–1 matrix-array transducer (composed of 3040 elements with 1–5 MHz). A non-foreshortened apical (A) four-chamber (ch), A3ch and A2ch were recorded with manual rotation. All echocardiographic images were obtained with a frame rate > 60 frames per second.

### Computed tomography

Acquisition was performed using a dual-source CT (Somatom Force or Somatom Definition Flash, Siemens Healthcare, Forchheim, Germany). Retrospective ECG-gated spiral acquisition was applied, and kV was modulated to patient size and a vascular exam type. Dose modulated ECG-pulsing was employed with nominal tube current during the 0 to 40% window of the R-R interval, and tube current reduced to 20% of the nominal output for the remainder to reduce the radiation dose. Reference tube current was set at 150 mAs per rotation. The pitch was adapted to increase proportionally with higher heart rates. No beta blockers were administered prior to the scan. Reconstructions were made with a medium smooth kernel. In total 20 different reconstructions with a slice thickness of 1.5 mm and 1.0 mm overlap were made in each patient at every 5% of the R–R interval. The mean dose length product (DLP) was 362 mGy-cm (estimated effective dose 5 mSv, using a conversion factor of k = 0.017). A 65 ml bolus of iodinated contrast material (Iodixanol 320, Visipaque, GE Healthcare, Cork, Ireland) was administered through an antecubital vein followed by a 40 ml 70/30% saline/contrast medium bolus, both at 5 ml/s.

### Cardiovascular magnetic resonance imaging

Image acquisition was performed using a 1.5 T clinical MRI scanner (Discovery MR450, GE Medical Systems, Milwaukee, WI, USA) using a 32-channel phased-array cardiac surface coil. The imaging protocol consisted of black blood TSE aorta, 2D phase contrast images for pulse wave velocity measurements, SSFP for aortic distensibility measurements, contrast enhanced MR angiography and 4D flow CMR of the entire heart and aorta. The 4D flow CMR was acquired immediately after the bolus injection of 0.1–0.2 mmol/kg gadolinium-based contrast agent (Gadovist 1 mmol/ml, Bayer, Mijdrecht, The Netherlands). The 4D flow sequence has been described before [24]. In short the sequence was prescribed in axial plane, including the entire thorax in the field of view. The k-space was filled with variable-density Poisson-disc under sampling with acceleration factors of 1.8 × 1.8 (phase × slice) and the parallel imaging algorithm used was ESPIRiT. Typical scan parameters were: matrix 192 × 160 × 78, acquired resolution 2.1 × 1.8 × 2.8 mm, reconstructed resolution 2.1 × 1.8 × 1.4 mm, flip angle 15°, views per segment 4, bandwidth 63 kHz, number of reconstructed phases 20 per cardiac cycle, and a velocity encoded value set at 250 cm/s. Due to restricted scan time per patient no SSFP cine images were acquired*.*

### Image analysis

**Fig. 1:**
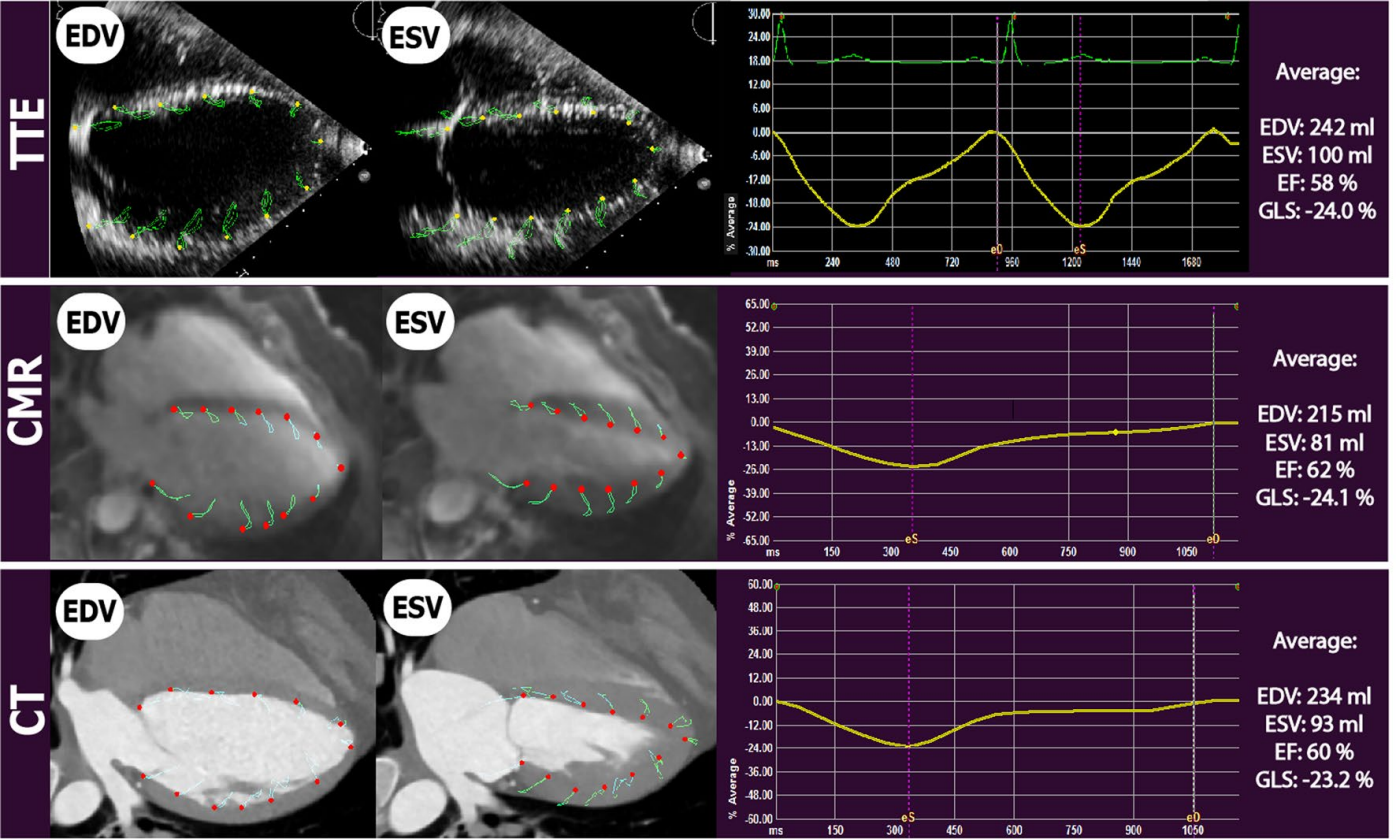
Left ventricular parameters by three different modalities in the same patient. *TTE* Transthoracic Echocardiography, *CT* Computed Tomography, *CMR* Cardiovascular Magnetic Resonance, *EDV* end-diastolic volume, *ESV* end-systolic volume, *EF* Ejection Fraction, *GLS* Global Longitudinal Strain. Yellow lines depict GLS during the cardiac cycle. *eS* end-systolic phase, *eD* End-diastolic phase

All images (CMR, CT and TTE) were analyzed by one observer (A.T.),who had 6 years of experience in cardiovascular imaging, in a random order and blinded to the results of the other image modalities. The TTE, CT, and CMR data was then re-measured by a second observer (S.Y.), who has one year of experience, blinded to the results of the first observer and to the corresponding measurements of the other modalities. For 2D TTE, speckle tracking analysis was performed using dedicated commercially available software (2D Cardiac Performance Analysis, Tomtec Imaging Systems). End-systolic and end-diastolic frames were identified manually; additionally the annulus and apex were identified manually in end-systole (Fig. 1). Subsequently, the software semi-automatically detected the end-diastolic and end-systolic myocardial contours. These contours were visually checked and corrected if necessary. This process was performed in all apical views (A2ch, A3ch, and A4ch).

All CT and CMR images were analyzed semi-automatically using commercially available software from Medis Medical Imaging Systems, Leiden The Netherlands. All images were loaded into the Medis Suite software (version: 3.1.16.6). A2ch, A3ch, and A4ch reconstructions were made from the 3D data sets using Medis 3D View (version: 3.1.18.1). For the CMR analysis only the magnitude images, which contain the anatomical data, of the 4D flow data set were used. All cardiac phases were included in the multi-plane reconstructions and endocardial contours were drawn manually at both end-diastole and end-systole using Medis QMass software (version: 8.1.30.4) where the papillary muscles and trabeculations were included in the LV lumen. Subsequently GLS, ejection fraction (EF), end-diastolic and end-systolic volume (EDV and ESV) were calculated using QStrain software (version: 3.1.16.6). Volumes were corrected for body surface area (BSA). BSA was calculated according to the Dubois formula [25]. In QMass LV contours were drawn manually and focused on adequate tracking of the myocardium for precise strain analysis. However, changes to the contours necessary for optimal tracking of the myocardium caused an underestimation of the ESV. Therefore, in order to provide data on the inter-modality variability of the volumes, a second set of separate endocardial contours had to be drawn for the measurement EDV, ESV and EF. The second trace of endocardial contours, drawn for the volumetric analysis, used standard anatomical landmarks (Supplemental Video 1). However, for adequate strain analysis the left ventricular outflow tract (LVOT) had to be excluded, and the first trace therefore started more apically in both end-systole and end-diastole, (Supplemental Video 2) to prevent highly positive segmental strain disturbing GLS measurement. For the inter-observer variability, twenty patients were chosen at random.

### Statistical analysis

The IBM SPSS® statistics 21.0 software was used to analyze the data. Continuous variables were presented as mean ± standard deviation (SD) or as median with an interquartile range. Categorical variables were presented as frequencies and percentages. We tested for normality by calculating Z-values of skewness and kurtosis, using the Shapiro–Wilk test and by visually assessing the data. For comparison of normally distributed continuous variables between two groups the student’s t-test was used. To quantify correlations the Pearson correlation test was applied. Inter-observer agreement between two investigators was assessed using Bland–Altman analysis [26]. The bias was defined as the mean absolute difference (i.e. the average absolute difference between two modalities). The limits of agreement between two measurements were determined as the mean of the difference ± 1.96 SD. Additionally, the coefficient of variation (COV) was provided to compare the dispersion of two variables. The COV was defined as the SD of the differences of two measurements divided by the mean of their means. The statistical tests were two sided and a p-value < 0.05 was considered significant.

## Results

Fifty-nine patients were included, of whom 37 men (63%). Their baseline characteristics are presented in Table 1. In 56 patients (95%) echo measurements could be performed, two patients did not undergo echocardiography due to organizational reasons and one patient was excluded because of insufficient image quality. In total 53 patients underwent a CT scan, of which one patient was excluded due to technical limitations, therefore 52 (88%) patients were included for CT analysis. In six patients no CT scan was done due to organizational reasons. In 48 patients (83%) 4D flow CMR was performed. In eleven patients 4D flow CMR was missing due to organizational reasons (scan time per patient was restricted and therefore 4D flow could not always be performed in all patients). The results of all measurements are presented per modality in Table 2. The results of the inter-modality agreement are presented in Table 3. All CT and MR scans were included. No scans (CT or CMR) were excluded because of insufficient image quality. A sensitivity analysis was conducted which showed that the there was no significant difference when only the patients who underwent all three modalities were considered (n = 39) (Tables 2 and 3).

**Table 1:** Baseline characteristics

Baseline characteristics	Median [IQR] (n = 59)
Age, years	34 [19]
Height, cm	180 [23]
Weight, kg	75 [19]
BMI, kg/m^2^	24 [3]
BSA, m^2^	1.9 [0.4]
SBP, mmHg	123 [17]
DBP, mmHg	79 [16]
Aortic valve	
Vmax, m/s	2.2 (1.6)
Peak Gradient, mmHg	19 (32)
AoI grade—none	12 (20)
AoI grade—moderate	34 (58)
AoI grade—severe	13 (22)
Sievers type*	
Type 0—lat	6 (10)
Type 0—ap	7 (12)
Type 1—LR	23 (49)
Type 1—RN	7 (12)
Type 1—LN	1 (2)
Type 2—LR/RN	7 (12)
Undetermined	2 (2)

Data are presented as median [IQR] or n(%)

*BMI* body-mass index, *BSA* body surface area, *SBP and DBP* systolic and diastolic blood pressure, *V*_*max*_ peak aortic valve velocity, *AoI* aortic valve insufficiency

*Valve type according to Sievers classification

**Table 2:** Left ventricular parameters per imaging modality

	All patients*			All three modalities completed^a^		
	CT (n = 52)	CMR (n = 48)	TTE (n = 56)	CT (n = 39)	CMR (n = 39)	TTE (n = 39)
GLS (%)	− 20 ± 3	− 21 ± 3	− 20 ± 3	− 21 ± 2	− 21 ± 3	− 20 ± 3
EF (%)	58 ± 6	54 ± 7	55 ± 6	58 ± 5	55 ± 7	55 ± 5
EDV (ml)	192 ± 65	203 ± 62	185 ± 64	183 ± 58	193 ± 62	180 ± 57
EDV/BSA (ml/m^2^)	99 ± 26	105 ± 26	95 ± 26	95 ± 24	100 ± 24	94 ± 24
ESV (ml)	82 ± 33	94 ± 34	83 ± 33	77 ± 28	87 ± 32	81 ± 29
ESV/BSA (ml/m^2^)	42 ± 13	48 ± 15	42 ± 15	40 ± 12	45 ± 13	42 ± 12

*EDV* end-diastolic volume, *ESV* end-systolic volume, *EF* ejection fraction, *GLS* global longitudinal strain, *BSA* body surface area, *TTE *transthoracic echocardiography, *CT* computed tomography, *CMR* cardiovascular magnetic resonance

*In this analysis all patients that completed 2 or more imaging modalities were considered

^a^In this sensitivity analysis data is shown when only patients are considered that completed all three imaging modalities Data are presented as mean ± standard deviation

**Table 3:** Inter-modality agreement

	All Patients					All three modalities				
	Pearson’s r^a^	Bias^b^	Mean difference	Lower LOA	Upper LOA	Pearson’s r^a^	Bias^b^	Mean difference	Lower LOA	Upper LOA
	CMR vs. TTE (n = 45)					CMR vs. TTE (n = 39)				
GLS (%)	0.67	3	− 2	− 7	3	0.69	2	− 2	− 6	3
EF (%)	0.61	4	0	− 11	10	0.62	4	0	− 10	11
EDV (ml)	0.84	31	16	− 53	85	0.86	28	13	− 50	75
ESV (ml)	0.82	17	8	− 32	47	0.85	15	6	− 28	40
	CT vs. TTE (n = 49)					CT vs. TTE (n = 39)				
GLS (%)	0.65	2	− 1	− 5	4	0.65	2	− 1	− 5	3
EF (%)	0.67	4	2	− 6	11	0.69	4	3	− 5	11
EDV (ml)	0.85	26	8	− 61	77	0.88	23	3	− 54	59
ESV (ml)	0.83	13	0	− 36	36	0.87	12	− 4	− 33	25
	CT vs. CMR (n = 42)					CT vs. CMR (n = 39)				
GLS (%)	0.62	2	1	− 4	6	0.56	2	1	− 5	6
EF (%)	0.68	5	3	− 8	14	0.56	5	3	− 8	14
EDV (ml)	0.93	19	− 11	− 56	35	0.93	18	− 10	− 54	33
ESV (ml)	0.90	14	− 11	− 40	19	0.90	13	− 10	− 38	18

*LOA* limit of agreement, *EDV* end-diastolic volume, *ESV* end-systolic volume, *EF* ejection fraction, *GLS* global longitudinal strain, *TTE* transthoracic echocardiography, *CT* computed tomography, *CMR* cardiovascular magnetic resonance

^a^all significant with a p < 0.001

^b^Defined as the mean absolute difference

### Left ventricular global longitudinal strain and ejection fraction

**Fig. 2:**
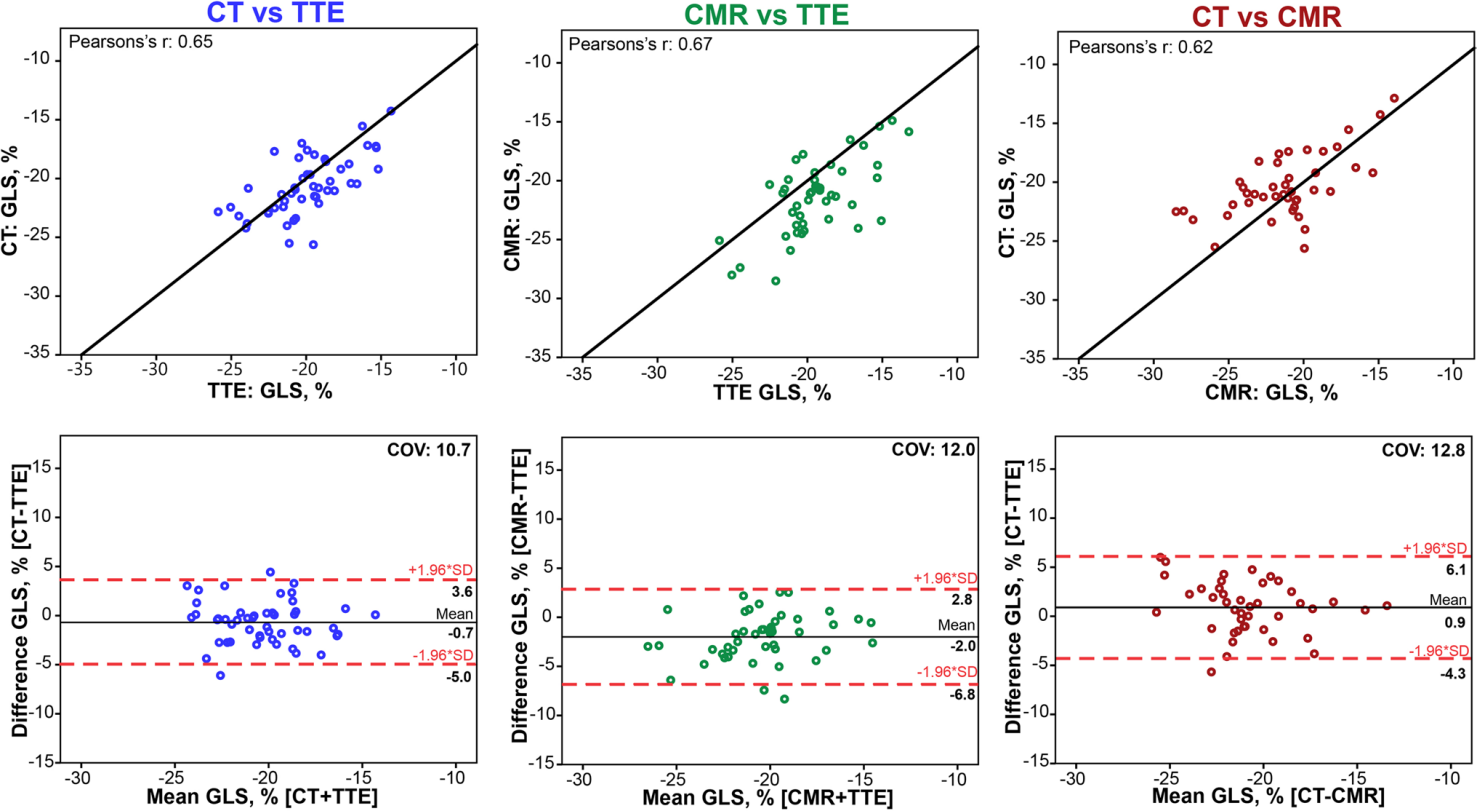
Inter-modality agreement for global longitudinal strain. Agreement between TTE Transthoracic Echocardiography, CT Computed Tomography, *CMR* Cardiovascular Magnetic Resonance, *GLS* global longitudinal strain. Bland–Altman plots and identity line (black) for CT versus TTE (blue) and CMR versus TTE (green) and CT versus CMR (red). Dashed red lines indicate ± 1.96 SD. *COV* coefficient of variation

**Fig. 3:**
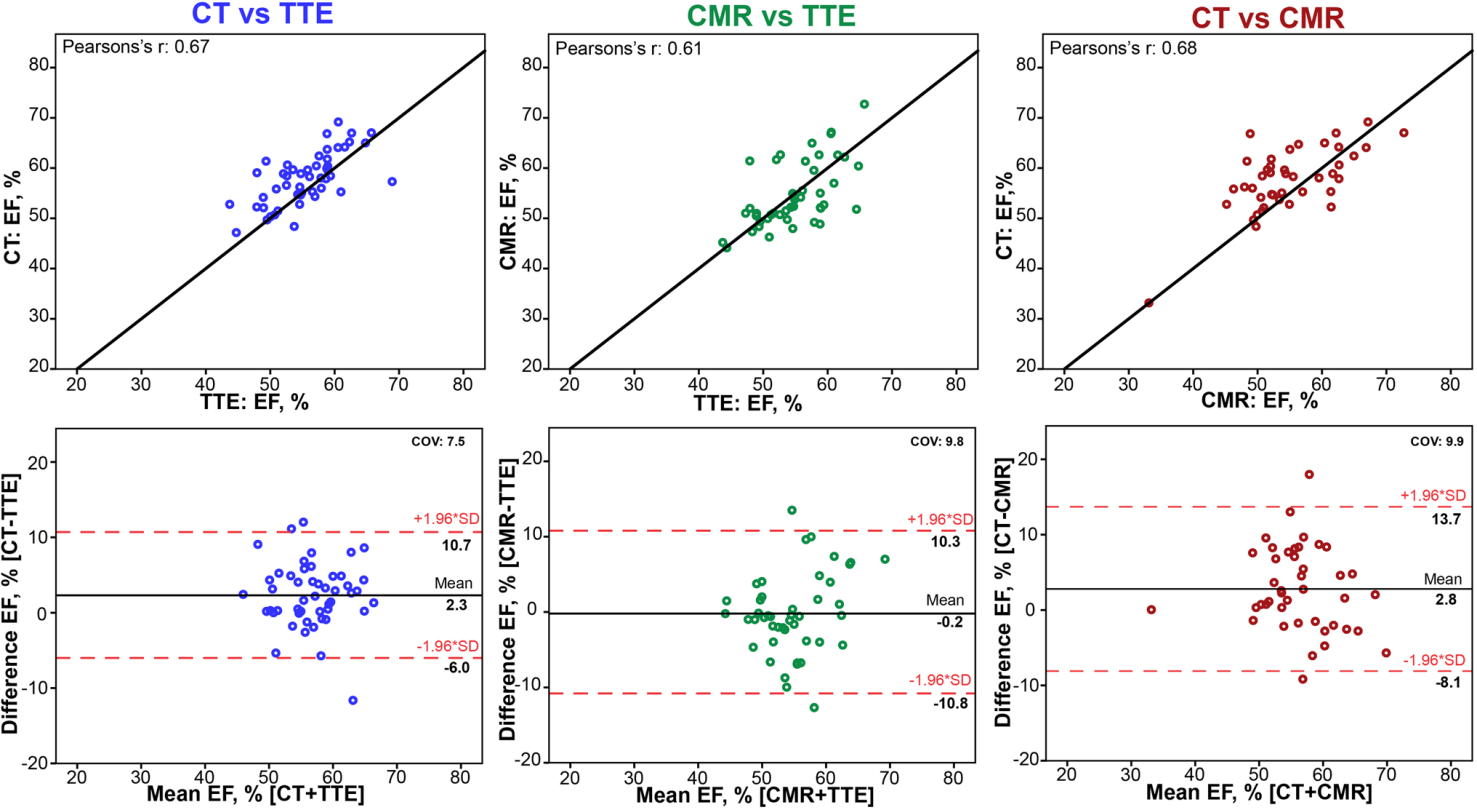
Inter-modality agreement for ejection fraction. Agreement between *TTE* Transthoracic Echocardiography, *CT* Computed Tomography, *CMR* Cardiovascular Magnetic Resonance, *EF* ejection fraction. Bland–Altman plots and identity line (black) for CT versus TTE (blue) and CMR versus TTE (green) and CT versus CMR (red). Dashed red lines indicate ± 1.96 SD. *COV* coefficient of variation

When comparing CMR and CT versus TTE, strong correlations (Table 3: Pearson’s r: 0.67, p < 0.001 and Pearson’s r: 0.65, p < 0.001 respectively) were found for GLS. However, especially CMR seemed to slightly overestimate GLS with a mean difference − 2% and a bias of 3% when compared to TTE (Fig. 2). The results of the EF measurements per modality are presented in Table 2; results for the agreement analysis of EF are shown in Table 3 and Fig. 3. Of all three modalities EF, measurement by CT yielded the highest mean EF: 58 ± 6%, where CMR yielded the lowest mean EF of the three modalities (54 ± 7%).

### Volume measurement

Correlations for LV end-diastolic volumes (Supplemental Fig. 4) were strong for both CMR and CT compared to TTE (Table 3, Pearson’s r: 0.84 and 0.85, both p < 0.001 respectively), where the mean difference was smallest between CT and TTE. As shown in Table 2, EDV measurements were larger on CMR (203 ± 62 ml) compared with TTE (185 ± 64 ml), which resulted in the largest bias of 31 ml and limits of agreement ranging between − 53 ml and 85 ml.

Correlations for ESV were comparable to those found for EDV (Supplemental Fig. 5). ESV measured by CMR and CT correlated strongly with TTE (Pearson’s r: 0.82 and 0.83 respectively, both p < 0.001). Here too CT compared best with TTE with a mean difference of − 0.3 ml (bias: 13 ml) versus 7.8 ml on average for CMR compared with TTE (bias: 17 ml).

### Inter-observer variability

Inter-observer variability was assessed for all three modalities; the results of the second observer agreement analysis for TTE are presented in Supplemental Fig. 6. Both for GLS and EF good inter-observer agreement was found (Pearson’s r: 0.70, p < 0.001 and 0.60, p = 0.006 respectively), and also for EDV and ESV (Pearson’s r: 0.96 and 0.90, p < 0.001 respectively). The relatively large mean difference for EF (− 9.4%) for TTE, was mainly driven by observer 1 overestimating both EDV (mean difference: 13.7 ml) and ESV, with a tendency towards a more significant overestimation of the ESV (mean difference: 27.2 ml) relative to the EDV. Inter-observer variability for CT is presented in Supplemental Fig. 7, where a strong correlation between observers was found for GLS with a mean difference of − 1.8% on average. Finally, inter-observer variability for CMR is presented in Supplemental Fig. 8, where a moderate correlation for GLS (Pearson’s r: 0.51, p = 0.023) was found.

## Discussion

In this prospective cohort study we demonstrated for the first time that the assessment of GLS is feasible using a feature-tracking algorithm on the magnitude images acquired by 4D flow CMR directly after gadolinium contrast. This opens the way for an integrative one-sequence approach in which both flow and functional information can be acquired simultaneously. Moreover, in this study functional LV parameters measured by CMR correlated well with 2D TTE, with a mean difference comparable to that found in other studies using ‘conventional’ SSFP cine CMR images [27–30]. On average GLS in our cohort of BAV patients was similar to that found using SSPF CMR images in a healthy adult population [31].

Although LV functional analysis by CT has been possible for a number of years there is limited data available on the value of CT in GLS assessment [16, 32–34]. CT has been shown to correlate closely with CMR and TTE for left ventricular assessment, [28] and more recently also for GLS analysis [29]. Additionally, studies have described good correlations between CT and TTE [16, 33]. Our study confirms these correlations with TTE and CMR for both GLS and EF. Furthermore, CT had the best reproducibility of all three modalities, reflected in the lowest coefficient of variation in the second-observer analysis. The observed overestimation of EF by CT compared to CMR could be explained by the fact that, especially on the long axis A3ch-view, papillary muscles are often difficult to discern resulting in a smaller ESV and subsequent high EF. Furthermore, unlike TTE and CMR, CT has the disadvantage of significant radiation exposure for the patient, since imaging of the complete cardiac cycle is necessary for GLS analysis.

CT and CMR correlated well both for GLS and EF. Based on the high spatial resolution CT could have been expected to outperform CMR, as CMR may require more observer interpretation in determining the endocardial contour. Indeed we observed a lower coefficient of variation for CT versus TTE (COV: 10.7) compared to CMR versus TTE (COV: 12.0) for both GLS and for EF (COV: 7.5 vs 9.8). Additionally, second observer analysis for CT showed a lower COV for all LV measurements.

A clear limitation of this study is the need for separate contours for the GLS and volumes, caused by the frequent inadequate tracking of the basal and mid and anterior septal segments by the feature-tracking algorithm on CT and CMR (Supplemental Fig. 9). Tracing the endocardial contour in the apical three chamber view from the mitral valve to the aortic valve orifice (Supplemental Video 1) often resulted in positive strain values in these segments, lowering the GLS. This could be resolved by placing the endocardial marker more apically (Supplemental Video 2) resulting in an underestimation of the EDV and ESV. A third video shows the same process for MR in apical three and 4 chamber views (Supplemental Videos 3 and 4 respectively). The difficulty here is that when abandoning the anatomical landmark there is no clear alternative, which introduces possible inter-observer variability. Although more time consuming we chose to draw a second endocardial tracing focusing on the volume quantification when this problem occurred. With regard to the post-processing process, we found the TTE workflow to be significantly less time intensive compared to CT and CMR, partly because with TTE the sonographer directly acquired the apical 2-, 3-, and 4-chamber views. Both with CT and CMR the observer had to create these views retrospectively. This may allow for more precise reconstruction and analysis, but it is also more time intensive as it increases workflow complexity, and creates a possible source of bias between observers. It has been shown that both observer experience and the software used for analysis can have a significant influence on the agreement for CMR (30, 31) as for TTE (32). And although outside the scope of this study we agree that user experience is an important factor in LV functional analysis. This is perhaps best reflected in the second observer analysis for TTE, where a small but consistent difference in EDV and ESV resulted in a systematically lower EF for the second observer. Another limitation is that we did not have SSFP CMR cine images available for these patients, which would have allowed to also compare 4D flow CMR with the ‘gold standard’ for volume quantification and feature-tracking strain analyses on SSFP images. We feel that part of the variation between CMR and the other modalities could be explained by the inferior spatial resolution of the magnitude image datasets. Furthermore, the standard deviation of GLS and EF in this patient cohort is small, as all patients had relatively preserved LV function. A future study could evaluate how this technique performs in patients with a reduced LV function.

## Conclusion

Feature-tracking GLS analysis is feasible using the magnitude images acquired by 4D flow CMR with adequate imaging quality. GLS measurement by CMR correlates well with CT and speckle-tracking 2D TTE. GLS analysis on 4D flow CMR allows for an integrative approach in which flow and functional data can be acquired in one sequence. Future studies should aim to validate these findings in a healthy control population, preferably compared with SSPF cine imaging.

## Electronic supplementary material

Below is the link to the electronic supplementary material.

Supplemental figure 1: Inter-modality agreement for end–diastolic volume. Agreement between transthoracic echocardiography (TTE), Transthoracic Echocardiography (TTE), Computed Tomography (CT) and Cardiovascular Magnetic Resonance (CMR) for end-diastolic volume (EDV). Bland-Altman plots and identity line (black) for CT versus TTE (blue) and CMR versus TTE (green) and CT versus CMR (red). Dashed red lines indicate ±1.96 SD. COV: coefficient of variation. (JPG 1,322 kb)

Supplemental figure 2: Inter-modality agreement for end-systolic volume. Agreement between transthoracic echocardiography (TTE), Computed Tomography (CT) and Cardiovascular Magnetic Resonance (CMR) for end-systolic volume (ESV). Bland-Altman plots and identity line (black) for CT versus TTE (blue), and CMR versus TTE (green) and CT versus CMR (red). Dashed red lines indicate ±1.96 SD. COV: coefficient of variation. (JPG 1,298 kb)

Supplemental figure 3: Inter-observer agreement for Transthoracic Echocardiography (TTE) (n = 20). Bland-Altman plots and identity line (black) for global longitudinal strain (GLS), ejection fraction (EF), end-diastolic volume (EDV) and end-systolic volume (ESV). Dashed red lines indicate ± 1.96 SD. COV: coefficient of variation. *All Pearson’s r’s are significant with a p < 0.01. (JPG 1445 kb)

Supplemental figure 4: Inter-observer agreement for Computed Tomography (CT) (n = 20). Bland-Altman plots and identity line (black) for global longitudinal strain (GLS), ejection fraction (EF), end-diastolic volume (EDV) and end-systolic volume (ESV). Dashed red lines indicate ± 1.96 SD. COV: coefficient of variation. *All Pearson’s r’s are significant with a p-vale of p < 0.001. (JPG 1421 kb)

Supplemental figure 5. Inter-observer agreement for Cardiovascular Magnetic Resonance (CMR) (n = 20). Bland-Altman plots and identity line (black) for global longitudinal strain (GLS), ejection fraction (EF), end-diastolic volume (EDV) and end-systolic volume (ESV). Dashed red lines indicate ±1.96 SD. COV: coefficient of variation. *All Pearson’s r’s are significant with a p-value < 0.05. (JPG 1,459 kb)

Supplemental figure 6. Separate contours for the volume and strain traces. contours as drawn for the volume trace (A and B) and the contours as drawn for the strain trace more apically (C and D). In both end-diastole (ED, A and C) and end-systole (ES, B and D). (JPG 277 kb)

Supplementary Video 1 (MP4 2831 kb)

Supplementary Video 2 (MP4 3411 kb)

Supplementary Video 3 (MP4 251 kb)

Supplementary Video 4 (MP4 214 kb)

## Data Availability

The data that support the findings of this study are available on request from the corresponding author [A.H.]. The data are not publicly available due to them containing information that could compromise individual research participant privacy.

## Abbreviations

LV: Left ventricular
GLS: Global longitudinal strain
TTE: Transthoracic echocardiography
CMR: Cardiovascular magnetic resonance
CT: Computed tomography
BAV: Bicuspid aortic valve
EF: Ejection fraction
EDV: End-diastolic volume
ESV: End-systolic volume measurements
SSFP: Steady state free-precession
DLP: Dose length product
2D: Two dimensional
3D: Three dimensional
4D: Four dimensional
COV: Coefficient of variation
LVOT: Left ventricular outflow tract
SD: Standard deviation
