# Supplementary figures and images for: Left ventricular global longitudinal strain in bicupsid aortic valve patients: head-to-head comparison between computed tomography, 4D flow cardiovascular magnetic resonance and speckle-tracking echocardiography
Source: Int J Cardiovasc Imaging. 2020 May 25;36(9):1771–80. doi: 10.1007/s10554-020-01883-9 (PMC7438282; doi:10.1007/s10554-020-01883-9)

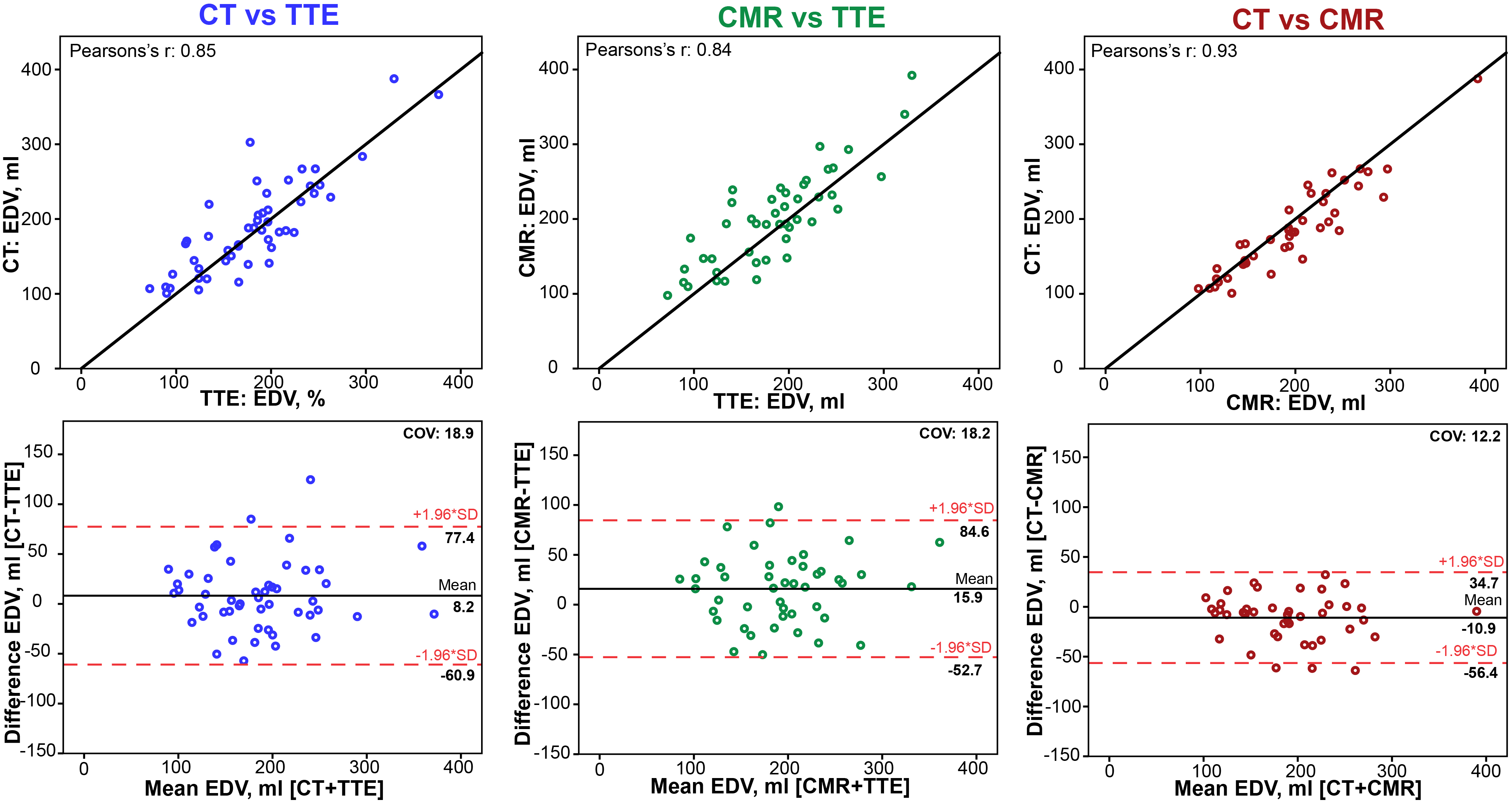

Supplement: Supplementary file 1 — Supplemental figure 1: Inter-modality agreement for end–diastolic volume. Agreement between transthoracic echocardiography (TTE), Transthoracic Echocardiography (TTE), Computed Tomography (CT) and Cardiovascular Magnetic Resonance (CMR) for end-diastolic volume (EDV). Bland-Altman plots and identity line (black) for CT versus TTE (blue) and CMR versus TTE (green) and CT versus CMR (red). Dashed red lines indicate ±1.96 SD. COV: coefficient of variation. (JPG 1,322 kb) [file 10554_2020_1883_MOESM1_ESM.jpg]

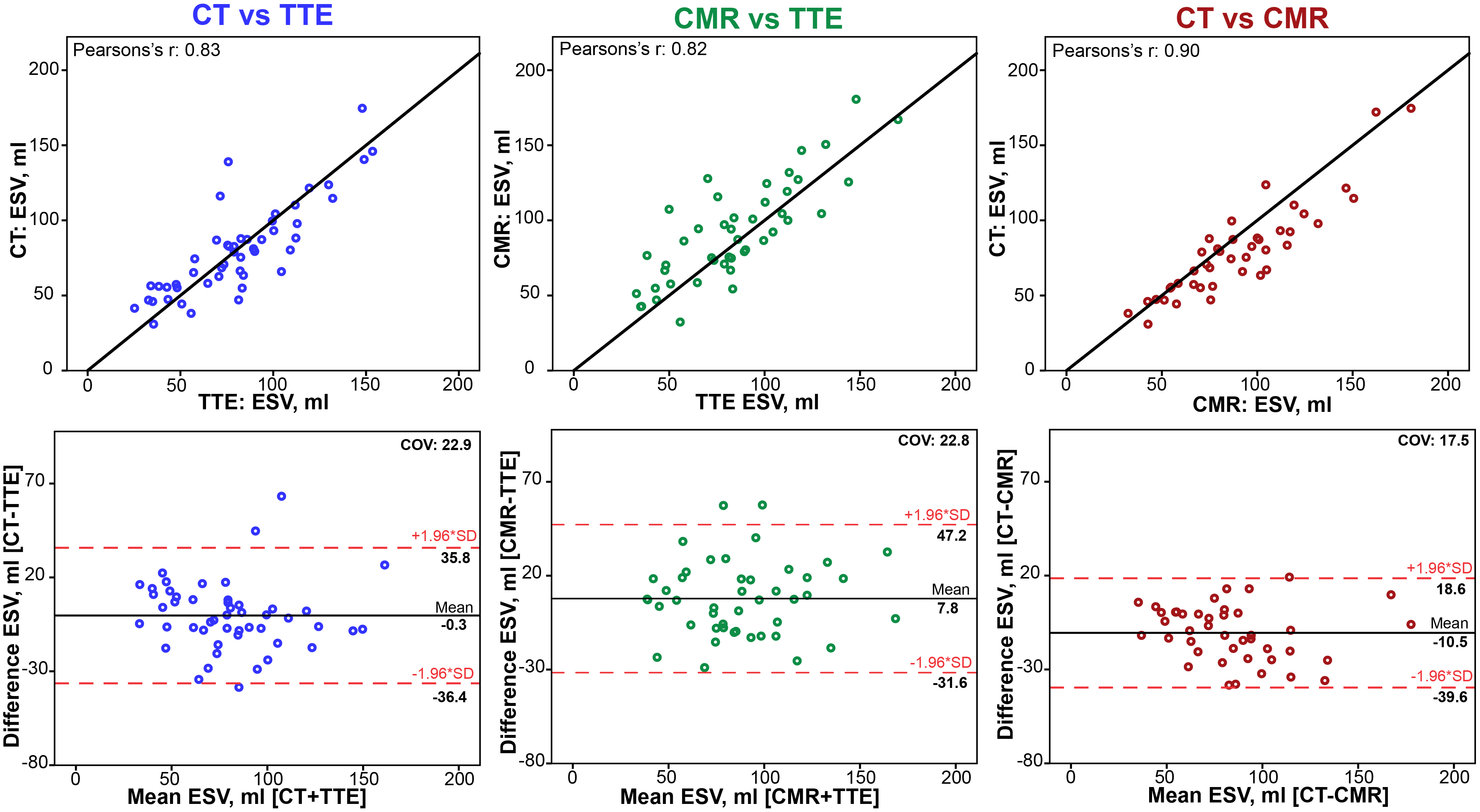

Supplement: Supplementary file 2 — Supplemental figure 2: Inter-modality agreement for end-systolic volume. Agreement between transthoracic echocardiography (TTE), Computed Tomography (CT) and Cardiovascular Magnetic Resonance (CMR) for end-systolic volume (ESV). Bland-Altman plots and identity line (black) for CT versus TTE (blue), and CMR versus TTE (green) and CT versus CMR (red). Dashed red lines indicate ±1.96 SD. COV: coefficient of variation. (JPG 1,298 kb) [file 10554_2020_1883_MOESM2_ESM.jpg]

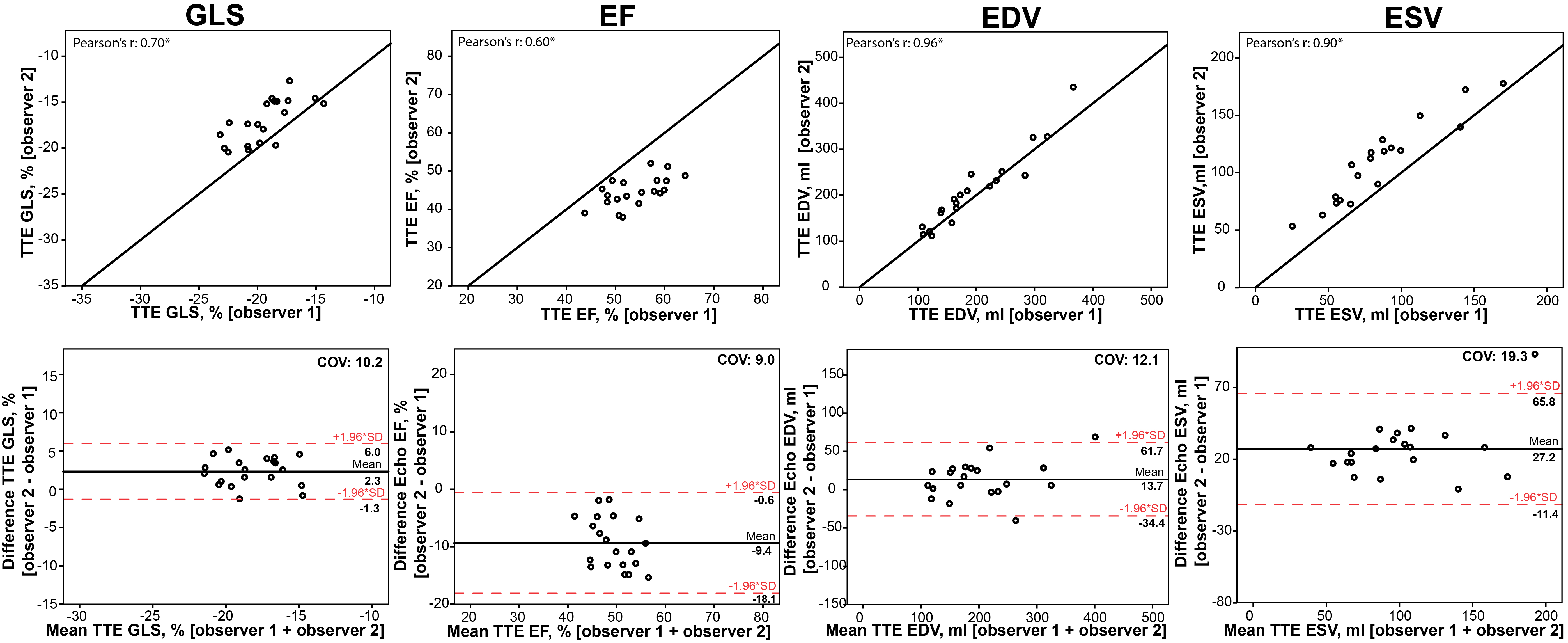

Supplement: Supplementary file 3 — Supplemental figure 3: Inter-observer agreement for Transthoracic Echocardiography (TTE) (n = 20). Bland-Altman plots and identity line (black) for global longitudinal strain (GLS), ejection fraction (EF), end-diastolic volume (EDV) and end-systolic volume (ESV). Dashed red lines indicate ± 1.96 SD. COV: coefficient of variation. *All Pearson’s r’s are significant with a p < 0.01. (JPG 1445 kb) [file 10554_2020_1883_MOESM3_ESM.jpg]

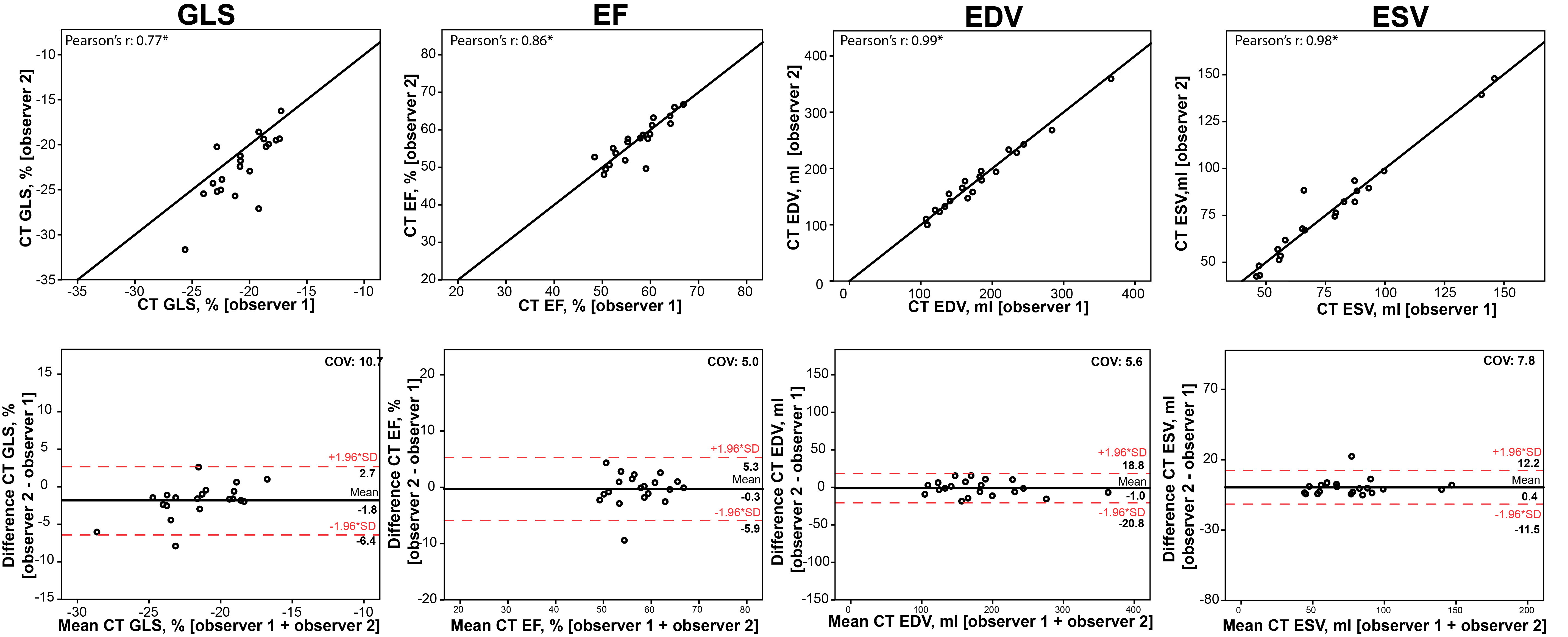

Supplement: Supplementary file 4 — Supplemental figure 4: Inter-observer agreement for Computed Tomography (CT) (n = 20). Bland-Altman plots and identity line (black) for global longitudinal strain (GLS), ejection fraction (EF), end-diastolic volume (EDV) and end-systolic volume (ESV). Dashed red lines indicate ± 1.96 SD. COV: coefficient of variation. *All Pearson’s r’s are significant with a p-vale of p < 0.001. (JPG 1421 kb) [file 10554_2020_1883_MOESM4_ESM.jpg]

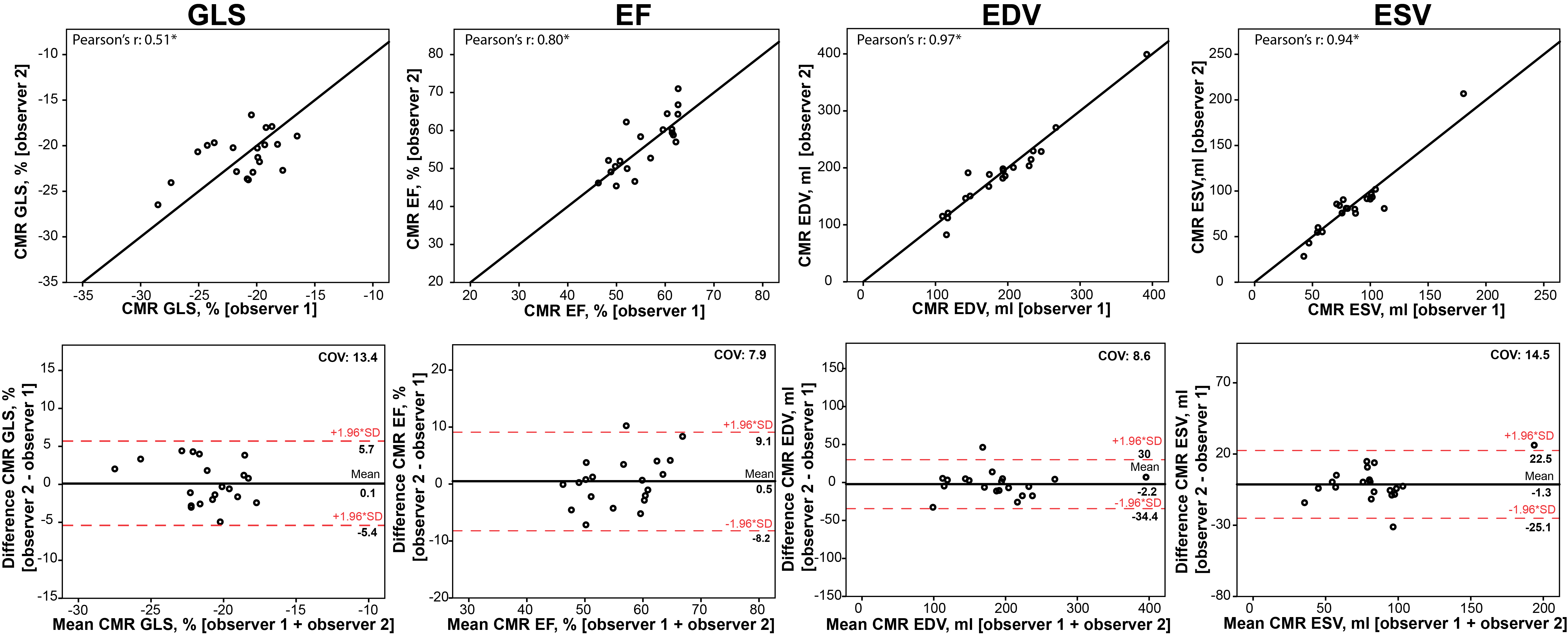

Supplement: Supplementary file 5 — Supplemental figure 5. Inter-observer agreement for Cardiovascular Magnetic Resonance (CMR) (n = 20). Bland-Altman plots and identity line (black) for global longitudinal strain (GLS), ejection fraction (EF), end-diastolic volume (EDV) and end-systolic volume (ESV). Dashed red lines indicate ±1.96 SD. COV: coefficient of variation. *All Pearson’s r’s are significant with a p-value < 0.05. (JPG 1,459 kb) [file 10554_2020_1883_MOESM5_ESM.jpg]

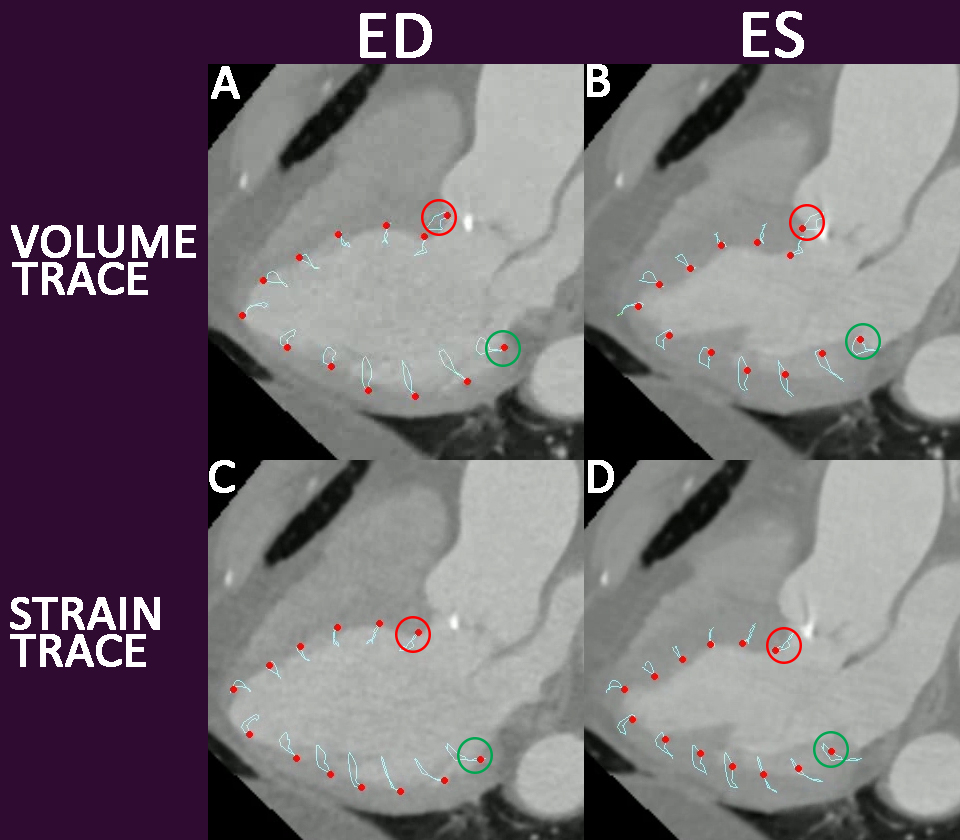

Supplement: Supplementary file 6 — Supplemental figure 6. Separate contours for the volume and strain traces. contours as drawn for the volume trace (A and B) and the contours as drawn for the strain trace more apically (C and D). In both end-diastole (ED, A and C) and end-systole (ES, B and D). (JPG 277 kb) [file 10554_2020_1883_MOESM6_ESM.jpg]
